# A possible association of baseline serum IL-17A concentrations with progression-free survival of metastatic colorectal cancer patients treated with a bevacizumab-based regimen

**DOI:** 10.1186/s12885-017-3210-z

**Published:** 2017-03-27

**Authors:** Emilie Lereclus, Mira Tout, Alban Girault, Nadine Baroukh, Morgane Caulet, Christophe Borg, Olivier Bouché, David Ternant, Gilles Paintaud, Thierry Lecomte, William Raoul

**Affiliations:** 1 Université François-Rabelais de Tours, CNRS, GICC UMR 7292, UFR de médecine, BP 3223, 10, boulevard Tonnellé, 37032 Tours Cedex 01, France; 2 CHRU de Tours, Department of Hepato-Gastroenterology and Digestive Oncology, Tours, France; 3 CHRU de Tours, Laboratory of Pharmacology-Toxicology, Tours, France; 4 INSERM, UMR 1098, Department of Medical Oncology, University Hospital, Besançon, France; 5 Department of Hepato-Gastroenterology and Digestive Oncology, University Hospital Robert Debré, Reims, France

**Keywords:** Bevacizumab, Vascular endothelial growth factor, Th17-related cytokines, IL-17 polymorphisms, Metastatic colorectal cancer, Survival analysis: score

## Abstract

**Background:**

Colorectal cancer is a major public health issue worldwide. Interleukin-17 (IL-17) and Th17 (T-helper cell type 17)-related molecules are involved in tumor development and in resistance to bevacizumab, an anti-vascular endothelial growth factor monoclonal antibody used in association with chemotherapy in metastatic colorectal cancer. Some studies have previously shown that IL-17A and IL-17F polymorphisms, respectively rs2275913 and rs763780, are associated with gastric or colorectal cancer risk. Here we aimed at studying the influence of IL-17A-related individual factors on overall survival and progression-free survival in patients with metastatic colorectal cancer treated with a bevacizumab-based chemotherapy.

**Methods:**

Pre-treatment serum biomarkers were retrospectively evaluated in 122 metastatic colorectal cancer patients treated by bevacizumab in combination with chemotherapy at 2-weeks intervals in a prospective cohort study (NCT00489697). The polymorphisms of IL-17A and IL-17F were analyzed by polymerase chain reaction - restriction fragment length polymorphism. Serum concentrations of Th17-related cytokines were measured by MultiPlex. The impact of individual parameters on overall survival and progression-free survival was assessed using multivariate Cox models.

**Results:**

High baseline IL-17A serum concentrations were significantly associated with shorter progression-free survival [*p* = 0.043]. Other baseline serum Th17-related cytokines and polymorphisms of IL-17 were not associated with overall survival or progression-free survival.

**Conclusions:**

In this ancillary study, baseline serum IL-17A concentration is the only Th17/IL-17 related factor that was significantly associated with the response of patients with metastatic colorectal cancer to bevacizumab. But this main significant result is highly dependent on one case which, if left out, weakens the data. Other clinical studies are required to confirm this association.

**Trial registration:**

NCT00489697. June 20, 2007.

## Background

Colorectal cancer is a major public health issue due to its frequency and its severity [1]. It is a major cause of death in the world [2]. The therapeutic arsenal against metastatic colorectal cancer was strengthened by the addition of monoclonal antibodies to chemotherapy. Bevacizumab (Avastin^®^ [3]) is a humanized IgG1 that binds to the vascular endothelial growth factor (VEGF), a well known pro-angiogenic growth factor [4] which favors tumors and metastasis. Bevacizumab is widely used in the treatment of patients with advanced colorectal cancer [5].

Though bevacizumab prolongs survival of patients with metastatic colorectal cancer, some individuals do not respond to treatment [6] and it is difficult to identify at an early stage who will benefit or not from this biopharmaceutical. Our team has recently showed that antigenic burden influences bevacizumab pharmacokinetics in patients with metastatic colorectal cancer [7]. In this study, bevacizumab pharmacokinetics was also influenced by baseline VEGF and CarcinoEmbryonic Antigen (CEA) concentrations and by the number of extra-hepatic metastases. These differences in bevacizumab pharmacokinetics between patients are relevant since bevacizumab concentrations are associated with progression-free (PFS) and overall survival (OS) of patients [7]. There is however a need to identify other early biomarkers that could be predictive of response to anti-VEGF biopharmaceuticals.

Several studies suggest that interleukin-17 (IL-17A or IL-17 or cytotoxic T-lymphocyte-associated protein 8) plays a major role in colorectal cancer progression [8–12] and in the resistance to anti-VEGF treatment in murine models [13] or chemotherapy [14, 15]. IL-17A is a pro-inflammatory cytokine that contributes to the pathogenesis of inflammatory and auto-immune diseases [16, 17] but that also seems to be highly associated with cancer progression [18, 19]. A major source of IL-17 is a lineage of T cells known as CD4^+^ T helper 17 cells (Th17 cells) which differ from the Th1 and Th2 subsets [20]. IL-17 is also secreted by other cell types of the immune system including lymphocytes NKT-17, γδT-17, CD8^+^ Tc17, polymorphonuclear neutrophils and intestinal Paneth cells [21, 22]. IL-17 homodimers signal through the complex formed by heterodimer IL-17 Receptor (R)A/IL-17RC.

Interleukin-17 F (IL-17F) is a recently described member of the IL-17 family with a great homology to IL-17A. IL-17F is mainly secreted by T CD4^+^ and γδT-17 lymphocytes and acts as homodimers or heterodimers with IL-17A [23]. It signals through the same receptors as IL-17A, with a better affinity to IL-17RC. However, its role and function in inflammation and cancer, a priori close to those of IL-17A, need to be further investigated. In colon cancer, these two cytokines could have in fact opposite effects since IL-17A favors cancer development and IL-17F appears to be a protective factor against tumorigenesis [18, 24]. Notably, IL-17A polymorphisms were associated with increased risk of colorectal cancer development when comparing patients to healthy controls in Iranian and Tunisian populations [25, 26]. Interestingly IL-17F polymorphisms was observed to be mainly protective in the same populations [25, 27].

To our knowledge, there is no data concerning serum Th17-related cytokines concentration or Il-17-related polymorphisms that may be predictive of the clinical impact of bevacizumab in metastatic colorectal cancer or be at least implicated in the prognosis of the disease, in this particular treatment schedule. The objective of the present study was therefore to evaluate the association of individual sources of variability related to IL-17 pathway with OS and PFS of patients with metastatic colorectal cancer treated with a bevacizumab-based regimen. In addition to biomarkers previously assessed, i.e. CEA, VEGF and bevacizumab concentrations [7], we studied the influence of baseline serum Th17-related cytokines concentrations (before bevacizumab treatment initiation) and of selected IL-17A (G197A, rs2275913) and IL-17F (T7488C, rs763780) polymorphisms on clinical outcomes.

## Methods

### Study design

This ancillary study is part of a French multicenter non-comparative, prospective, open-label, observational study (NCT00489697, registration first received June 20, 2007). The study is also registered as ID number INCA06-FT/STIC-AVASTIN. The main study was designed to evaluate the usefulness of hepatic contrast-enhanced ultrasound sonography as predictor of response to bevacizumab-based chemotherapy in patients with metastatic colorectal cancer. Briefly, patients enrolled between January 2007 and December 2010 received 5 mg/kg bevacizumab intravenously with two-week intervals in combination with chemotherapy. Tumor response was assessed by RECIST criteria (response evaluation criteria in solid tumors [28]) using spiral Computed Tomography (CT) before treatment and at day 60 after the beginning of the treatment. The end of follow-up was December 2012. Finally, 122 patients received at least the four bevacizumab infusions.

This study was designed in accordance with legal requirements and the Declaration of Helsinki and was approved by the ethics committee of Tours University Hospital, France. All patients gave written informed consent to participate in this study, including constitutional genetic analyses. Eligible patients (18–80 years old) had histologically confirmed metastatic colorectal cancer with at least one hepatic metastasis detected by ultrasonography, a life expectancy of more than two months, a World Health Organization performance status of two or less and were mostly treated as first line treatment with a bevacizumab-based chemotherapy.

### Polymerase chain reaction-restriction fragment length polymorphism (PCR-RFLP)

Genomic DNA was extracted from peripheral blood leukocytes. Patients were genotyped using specific primers. 100 ng/μl DNA was amplified with Taq Polymerase solution (Life Technologies, Saint-Aubin, France), including MgCl_2_ (2.5 mM) and dNTP (5 nM). PCR reactions were performed in 35 cycles of denaturation at 95 °C for 5 s and primer annealing and extension at 60 °C for 45 s.

IL-17A rs2275913 (G197A) sequence was amplified with the following primers previously validated by Wu et al. [29]:

Forward 5′-AAC AAG TAA GAA TGA AAA GAG GAC ATG GT-3′.

Reverse 5′-CCC CCA ATG AGG TCA TAG AAG AAT C-3′.

Il-17F rs763780 (A7488G) was amplified with the following primers previously validated by Wrobel et al. [30]:

Forward 5′-ACC AAG GCT GCT CTG TTT CT-3′.

Reverse 5′-GGT AAG GAG TGG CAT TTC TA-3′.

PCR products were digested overnight at 37 °C with FastDigest *XagI* (*EcoNI,* Fermentas, Illkirch, France) for rs2275913 variant and with FastDigest *NlaIII* (*Hin1II,* Fermentas) for rs763780 variant. The fragments were then separated on 8% TBE polyacrylamide gels (Invitrogen) and visualized after staining with ethidium bromide under UV light. The visualized DNA products for rs2275913 were 102 bp (AA genotype), 102 + 68 + 34 bp (AG genotype) and 68 + 34 bp (GG genotype). The visualized products for rs763780 were 412 bp (GG genotype), 412 + 288 + 124 bp (AG genotype) and 288 + 124 bp (AA genotype).

### Multiplex

Baseline concentrations of Th17-related cytokines were measured in serum samples using multiplex assay following manufacturer’s protocol (Bio-Plex Pro™ Human Th17 Cytokine Assays, Bio-Rad, Marnes-la-Coquette, France). This magnetic bead–based immunoassay allows to measure 15 proteins involved in the Th17 immune response pathway: IL-1β, IL-4, IL-6, IL-10, IL-17A, IL-17F, IL-21, IL-22, IL-23, IL-25, IL-31, IL-33, IFNγ, TNF-α and sCD40L.

### Survival analysis

Overall survival (OS) was defined as the time from first bevacizumab infusion to death from any cause, and progression-free survival (PFS) was defined as the time from first bevacizumab infusion to documented disease progression. OS and PFS were assessed using Cox semi-parametric (univariate and multivariate) proportional hazard regression models. This analysis aimed at testing and quantifying the association of circulating Th17-related cytokines concentrations (IL-1β, IL-4, IL-6, IL-10, IL-17A, IL-17F, IL-21, IL-22, IL-23, IL-25, IL-31, IL-33, IFNγ, TNF-α and sCD40L) and gene polymorphisms (IL-17A, GG vs AA + AG carriers; IL-17F, AA vs AG) as covariates of Cox models. The association between these covariates and OS or PFS, was assessed using forward and backward stepwise procedures. The relationship between survival and the following variables was also examined: age at inclusion, gender, bevacizumab trough concentrations before the second injection (C_trough_; <15.5 mg/L (median) vs ≥15.5 mg/L), and baseline concentrations of VEGF and CEA. For interleukin concentration measurements, values below the limit of detection (LOD) were replaced by the LOD value. Hazard ratios (HR) and their corresponding 95% confidence intervals (95% CI) were estimated. The association between covariates and survival functions was tested using Wald’s test, with alpha risk (*p*) <0.05. Cox survival analyses were performed using R Software version 3.2.2 (The R Foundation for Statistical Computing, Vienna, Austria).

## Results

### Demographic data and clinical features (Table 1)

One hundred and thirty patients were included but we analyzed only the 122 patients who received at least the four bevacizumab infusions. Median age was 65 years and patients were predominantly men (77%). The majority of patients were diagnosed for a colon cancer (72%), surgically resected (63%) with hepatic metastases only (58%). Bevacizumab associated with FOLFIRI was mostly used as first-line treatment (66%). The Table 1 below sums up the demographic data of patients and baseline measures (notably CEA, VEGF and bevacizumab concentrations), previously assessed [7].

**Table 1:** Demographic data of patients and baseline measures (*n* = 122)

Baseline patients’ characteristics (*n* = 122)		
Age, years		65 [58–72]
Weight, kg		68 [60–77]
Height, cm (*n* = 121)		170 [163–174]
Body surface area, m^2^ (*n* = 121)		1.75 [1.64–1.93]
Carcinoembryonic antigen, μg/L (*n* = 111)		68.6 [11.8–271.3]]
Vascular endothelial growth factor, ng/L (*n* = 119)		169.6 [61.9–334.5]
C_trough_ bevacizumab, mg/L		15.5 [11.7–22.8]
Time to progression, months		10.6 [7.4–16.1]
Follow-up period, months		23.9 [14.7–35.3]
Gender	male	77 (63%)
	female	45 (37%)
World Health Organization Performance Status (*n* = 116)	0	63 (52%)
	1	51 (42%)
	2	2 (2%)
Primary tumor site	Colon	85 (70%)
	Rectal	37 (30%)
Combined chemotherapy	FOLFIRI	81 (66%)
	FOLFOX	21 (17%)
	LV5FU2	8 (7%)
	Others	12 (10%)

Results are expressed as median [interquartile range] for continuous variables or number (%) for categorical variables.

*Abbreviations*: *n* number of patients, *FOLFIRI* oxaliplatin, 5-fluorouracil, and leucovorin, *FOLFOX* irinotecan, 5-fluorouracil, and leucovorin, *LV5FU2* leucovorin and 5-fluorouracil

### Polymorphisms

For rs2275913 IL-17A polymorphism, genotypes were distributed as follows (Table 2): homozygous AA 10.9%, heterozygous AG 51.1% and homozygous GG 38%. For rs763780 IL-17F polymorphism, genotypes were distributed as follows (Table 2): homozygous AA 92.6% and homozygous AG 7.4%. There were no homozygous GG for this polymorphism in our study.

**Table 2:** Distribution and frequencies of IL-17A and IL-17F genotypes in metastatic colorectal cancer patients (*n* = 122)

Polymorphisms	n	(%)
IL-17A (rs2275913)		
AA	14	11,47
AG	63	51,65
GG	45	36,88
IL-17F (rs763780)		
AA	116	95,08
AG	6	4,92
GG	0	0

There was no association between the genotypes and serum cytokine levels in this cohort.

### Cytokine concentrations

**Fig. 1:**
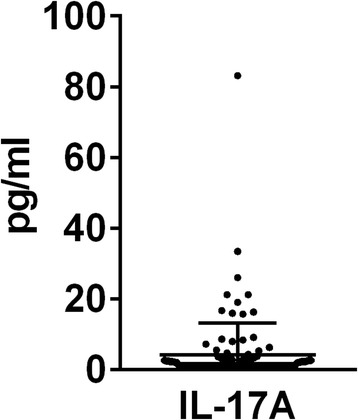
Distribution of baseline serum IL-17A concentrations of patients with metastatic colorectal cancer

The concentrations of cytokines involved in the Th17 immune response pathway are summarized in Table 3. IFNγ was detected in none of the included patients. The distribution of IL-17A concentrations was shown in Fig. 1 since it was the only Th17-related cytokine associated with PFS (see below the detailed Cox multivariate analysis, Table 5). For this cytokine, the range of values was similar to previous studies [11, 31].

**Table 3:** Baseline concentrations of serum Th17-related cytokines (*n* = 122)

Cytokine	Median concentration (pg/ml)	Range
IL-1β	0.24	[0.24–2.52]
IL-4	1.33	[1.33–95.56]
IL-6	13.08	[1.65–684.30]
IL-10	1.99	[1.99–70.69]
IL-17A	1.20	[1.20–83.17]
IL-17F	3.04	[3.04–128.41]
IL-21	8.97	[8.97]
IL-22	3.88	[3.88–21.09]
IL-23	7.35	[7.35–133.54]
IL-25	1.00	[1.00–1.63]
IL-31	3.87	[3.87–1089.37]
IL-33	4.18	[4.18–391.39]
IFNγ	2.54	[2.54]
sCD40L	493.64	[2.41–4580.84]
TNFα	0.75	[0.57–11.32]

There was no association between chemotherapy backbone and outcomes or Th17-related cytokines levels in this cohort.

### Overall survival (Table 4)

Median OS was 23.9 months (95% CI, 20.6–31.3 months). The univariate Cox analysis identified high baseline VEGF, IL-31 and CEA concentrations, and low bevacizumab C_trough_ as risk factors of death. In the multivariate analysis, high baseline CEA concentrations (HR = 1.15; 95% CI, 1.04–1.27; *P* = 0.0064) and low bevacizumab C_trough_ (<15.5 mg/L; HR = 1.89; 95% CI, 1.20–2.97; *P* = 0.0063) were independent risk factors of death.

**Table 4:** Cox regression analyses of potential factors associated with overall survival in patients with metastatic colorectal cancer (*n* = 122)

	HR	95% CI	*p*
Univariate analysis: Covariate			
Gender (female)	0.68	0.44–1.06	0.092
Age, years	1.01	0.99–1.03	0.55
Vascular endothelial growth factor, μg/L	2.09	1.10–3.99	0.025
C_trough_ bevacizumab <15.5 mg/L	2.34	1.52–3.58	9.9 × 10^**−5**^
Log(carcinoembryonic antigen), μg/L	1.21	1.10–1.34	9.8 × 10^**−5**^
IL17A polymorphism (A carriers)	0.86	0.56–1.30	0.47
IL17F polymorphism (AG)	1.80	0.87–3.73	0.11
IL-1β, pg/mL	1.23	0.69–2.21	0.48
IL-4, pg/mL	1.00	0.99–1.01	0.96
IL-6, pg/mL	1.00	0.99–1.00	0.49
IL-10, pg/mL	1.00	0.99–1.02	0.61
IL-17A, pg/mL	1.01	0.99–1.03	0.22
IL-17F, pg/mL	0.99	0.97–1.01	0.34
IL-22, pg/mL	1.07	0.99–1.15	0.057
IL-31, pg/mL	1.00	1.00–1.004	0.019
IL-33, pg/mL	1.00	0.99–1.00	0.95
sCD40L, pg/mL	1.00	0.86–1.06	0.83
Tumor necrosis factorα, pg/mL	0.97	0.86–1.09	0.61
Multivariate analysis: Covariate			
C_trough_ bevacizumab <15.5 mg/L	1.88	1.19–2.97	0.0064
Log(CEA), μg/L	1.15	1.04–1.28	0.0057

*Abbreviations*: *HR* hazard ratio, *95% CI* 95% confidence interval, *C*_*trough*_ trough concentration of bevacizumab before second infusion

### Progression-free survival (Table 5)

Median PFS was 10.6 months (95% CI, 9.6–12.4 months). The univariate analysis showed that high baseline IL-17A, VEGF and CEA concentrations, and low bevacizumab C_trough_ were risk factors of progression. In the multivariate analysis, high baseline concentrations of IL-17A (HR = 1.02; 95% CI, 1.00–1.04; *P* = 0.043) and VEGF (HR = 2.34; 95% CI, 1.31–4.20; *P* = 0.0041), and low bevacizumab C_trough_ ((<15.5 mg/L; HR = 1.72; 95% CI, 1.17–2.53; *P* = 0.0059) were independent risk factors of progression. In this new set of analysis and considering the selected Th17/IL-17A-related factors, VEGF and bevacizumab concentrations remain independent risks factors as recently described by Caulet et al. [7].

**Table 5:** Cox regression analyses of potential factors associated with progression-free survival in patients with metastatic colorectal cancer (*n* = 122)

	HR	95% CI	*p*
Univariate analysis: Covariate			
Gender (female)	0.70	0.47–1.03	0.07
Age, years	1.01	0.99–1.02	0.52
Vascular endothelial growth factor, μg/L	2.54	1.44–4.48	0.0013
C_trough_ bevacizumab <15.5 mg/L	1.88	1.28–2.74	0.0011
Log(carcinoembryonic antigen), μg/L	1.10	1.02–1.19	0.017
IL-17A polymorphism (A carriers)	0.80	0.55–1.17	0.26
IL-17F polymorphism (AG)	1.27	0.64–2.52	0.49
IL-1β, pg/mL	1.34	0.77–2.33	0.31
IL-4, pg/mL	1.00	0.99–1.01	0.74
IL-6, pg/mL	1.00	0.59–0.55	0.55
IL-10, pg/mL	1.01	0.99–1.02	0.49
IL-17A, pg/mL	1.02	1.00–1.04	0.026
IL-17F, pg/mL	1.00	0.99–1.02	0.65
IL-22, pg/mL	1.03	0.95–1.10	0.49
IL-31, pg/mL	1.00	0.99–1.00	0.089
IL-33, pg/mL	1.00	0.99–1.00	0.13
sCD40L, pg/mL	1.00	0.99–1.00	0.8
Tumor necrosis factorα, pg/mL	1.10	0.99–1.21	0.067
Multivariate analysis: Covariate			
Vascular endothelial growth factor, μg/L	2.34	1.31–4.20	0.0041
C_trough_ bevacizumab <15.5 mg/L	1.72	1.17–2.53	0.0059
IL-17A, pg/mL	1.02	1.001–1.04	0.043

*Abbreviations*: *HR* hazard ratio, *95% CI* 95% confidence interval, *C*_*trough*_ trough concentration of bevacizumab before second infusion

**Fig. 2:**
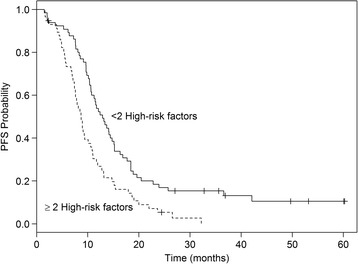
Kaplan–Meier plots. Progression-free survival according to the score calculated by baseline concentrations of plasma VEGF and serum IL-17A and bevacizumab C_trough_. *P* = 0.0006 by log-rank test

We next assessed whether the combination of the independent factors identified by multivariate Cox analysis would increase the predictive ability of each individual factor for PFS. A risk score was calculated for each patient, by summing-up the number of high-risk factors defined as IL-17A and VEGF concentrations above the respective median values, and bevacizumab C_trough_ below the median. Median PFS was significantly longer in patients with <2 high-risk factors compared to those with ≥2 high-risk factors (12.9 vs 8.7 months, *P* = 0.0006 by log-rank test; Fig. 2).

## Discussion

Drugs that target the VEGF pathway, associated with chemotherapy backbone, have a significant impact in metastatic colorectal cancer therapy . Bevacizumab in combination with chemotherapy has been proven to extend OS in first-line therapy [5] and in second-line therapy [32]. A pooled analysis from seven phase II and III (3763 patients) demonstrated that the use of bevacizumab with chemotherapy was associated with statistically significant benefits in survival outcomes, compared to chemotherapy alone, in the treatment of metastatic colorectal cancer [33].

According to the literature, IL-17A is involved in colorectal cancer progression, in cancer resistance and tumor escape following anti-VEGF-based therapy [11, 13, 15, 18, 34]. In addition, IL-17A is a factor that regulates T cell plasticity and might also promote antitumor immunity [35]. In syngeneic tumor pre-clinical models, Chung et al. [13] well described how IL-17A is implicated in a network that favors tumor escape following anti-VEGF treatment, notably via an IL17/G-CSF (granulocyte colony-stimulating factor)/Bv8 (prokineticin-2) alternative pathway initiated by Th17 infiltration. To study the relevancy of their pre-clinical findings, they investigated notably tissue sections from patients with colorectal cancer. Aa strong correlation of IL-17A–positive lymphocytes and Bv8-expressing polymorphonuclear granulocytes was found. In our ancillary study, we therefore sought to investigate the influence of IL-17A-related individual factors on OS and PFS in patients with metastatic colorectal cancer treated with bevacizumab mainly associated with FOLFIRI regimen.

In addition to the risk factors of progression reported by Caulet et al. [7] and regarding this cohort of patients with advanced colorectal cancer treated with a bevacizumab-based regimen, serum IL-17A baseline concentrations were identified as an independent factor and no influence of other Th17/Il-17A-related markers was observed.

To our knowledge, this is the first report that investigates IL-17A and its related factors as potential biomarkers of response of bevacizumab-based regimen in advanced colorectal cancer. However this study has some limitations since there is no control arm without bevacizumab therapy, which means that our findings have a prognostic role and other studies are needed to validate a predictive value. We can not conclude on whether our finding is attributable to bevacizumab alone or in combination with backbone chemotherapy. A recent systematic meta-analysis showed that there was no statistically significant difference in efficacy when bevacizumab is used with both irinotecan- or oxaliplatin-based regimens [36].

Some reports indicated that IL-17A could initiate and favour the tumoral progression at early colorectal cancer stages [8, 37] so it could be interesting to evaluate the impact of IL-17A concentrations at this time in future prospective studies including bevacizumab treatment. Wang et al. measured IL-17A concentrations in serum of patients with colorectal cancer that did not receive bevacizumab infusions [31]. They showed that significantly higher IL-17A concentrations were found at early colorectal cancer stages (I/II) concomitantly with increased concentrations of Il-23, another interleukin linked to Th17 differentiation, compared to control healthy donors. Tseng et al. also found that high IL-17A concentrations at stage II were associated with shorter disease-free survival in an independent analysis [11]. Moreover, retrospectively and irrespective of bevacizumab therapy, Tosolini et al. [38] showed that colorectal cancer patients with high expression of the Th17 genes cluster, analyzed from frozen specimens, had a poor prognosis and this result was confirmed with the quantification of IL-17-positive cells in situ. To date, only one team reported that high IL-17A expression, compared to expression in adenoma and non-tumor tissue, is an independent favorable prognostic factor that predicts overall survival of colorectal cancer by quantifying RNA expression in tissues and IL-17A immunohistochemical levels [39]. All the other publications reported that IL-17A high expression (RNA, immunohistochemistry and/or serum concentration) is a negative prognostic marker of colorectal cancer progression [8, 9, 38, 40].

The rs2275913 polymorphism can promote production of high levels of IL-17A and lead to enhanced IL-17-mediated immune responses. The rs763780 polymorphism leads to a Histidine-to-Arginine substitution and therefore antagonizes the function of wild-type IL-17F [41]. In our study we observed no influence of IL-17A and IL-17F polymorphisms on OS and PFS of our bevacizumab-treated group of patients with metastatic colorectal cancer.

Among individual Th17-related potential biomarkers, only baseline serum IL-17A concentrations are herein associated with PFS in patients with metastatic colorectal cancer treated with a bevacizumab-based chemotherapy. We can extrapolate that the clinical response could be influenced by or associated with other individual Th17-related biomarkers in larger panels or at early stages of the disease, e.g. IL-6 and IL-23 as markers of Th17 maturation or IL-17 and TNF-α as the main secreted factors. The fact that Il-17A was the only significant Th17-related factor associated with PFS must be reproducibly analyzed in other prospective studies. Moreover, it is important to distinguish the impact of IL-17A and the infiltration of Th17 cells. A recent meta-analysis following Prisma guidelines emphasizes the fact that high IL-17A quantities were correlated with poor prognosis in cancer and notably in colorectal cancer but the impact of high Th17 cell frequencies is very controversial and depends on the cancer type [42].

## Conclusions

To conclude, we think that evaluation of serum IL-17A concentration alone is probably not clinically relevant and IL-17A concentration should be assessed as an additional risk factor, along with baseline VEGF concentration and bevacizumab C_trough_. It could help to stratify patients with metastatic colorectal cancer receiving bevacizumab as first-line treatment according to their risk of disease progression (see Fig. 2). Considering the range of hazard ratios and *p* values in the Cox regression multivariate analysis, serum Il-17A concentrations play a significant role in the risk score but to a lesser degree than plasma VEGF and bevacizumab trough concentrations. But this significant result is highly dependent on one case which, if left out, weakens the data. Therefore the association should be confirmed in other clinical studies. And the determination of IL-17A concentrations at baseline, along with baseline VEGF concentrations and bevacizumab C_trough_, could also be embedded in the design strategy for future prospective clinical trials. In a near future, it would be interesting to target IL-17A, concomitantly or sequentially to VEGF inhibition, to overcome resistance to anti-VEGF-based therapy in metastatic colorectal cancer.

## Abbreviations

95% CI: 95% confidence interval
CEA: Carcinoembryonic antigen
C_trough_: Trough concentration of bevacizumab before second infusion
G-CSF: Granulocyte colony-stimulating factor
HR: Hazard ratio
IL-17A: Interleukin-17 A
OS: Overall survival
PFS: Progression-free survival
VEGF: Vascular endothelial growth factor
